# A Single Amino Acid Substitution in the Core Protein of West Nile Virus Increases Resistance to Acidotropic Compounds

**DOI:** 10.1371/journal.pone.0069479

**Published:** 2013-07-18

**Authors:** Miguel A. Martín-Acebes, Ana-Belén Blázquez, Nereida Jiménez de Oya, Estela Escribano-Romero, Pei-Yong Shi, Juan-Carlos Saiz

**Affiliations:** 1 Departamento de Biotecnología, Instituto Nacional de Investigación y Tecnología Agraria y Alimentaria (INIA), Madrid, Spain; 2 Wadsworth Center, New York State Department of Health, Albany, New York, United States of America; Metabiota, United States of America

## Abstract

West Nile virus (WNV) is a worldwide distributed mosquito-borne flavivirus that naturally cycles between birds and mosquitoes, although it can infect multiple vertebrate hosts including horses and humans. This virus is responsible for recurrent epidemics of febrile illness and encephalitis, and has recently become a global concern. WNV requires to transit through intracellular acidic compartments at two different steps to complete its infectious cycle. These include ***fusion*** between the ***viral envelope*** and the ***membrane*** of endosomes during viral entry, and virus maturation in the *trans*-Golgi network. In this study, we followed a genetic approach to study the connections between viral components and acidic pH. A WNV mutant with increased resistance to the acidotropic compound NH_4_Cl, which blocks organelle acidification and inhibits WNV infection, was selected. Nucleotide sequencing revealed that this mutant displayed a single amino acid substitution (Lys 3 to Glu) on the highly basic internal capsid or core (C) protein. The functional role of this replacement was confirmed by its introduction into a WNV infectious clone. This single amino acid substitution also increased resistance to other acidification inhibitor (concanamycin A) and induced a reduction of the neurovirulence in mice. Interestingly, a naturally occurring accompanying mutation found on prM protein abolished the resistant phenotype, supporting the idea of a genetic crosstalk between the internal C protein and the external glycoproteins of the virion. The findings here reported unveil a non-previously assessed connection between the C viral protein and the acidic pH necessary for entry and proper exit of flaviviruses.

## Introduction

West Nile virus (WNV) is a member of the *Flaviviridae* family that belongs to the *Flavivirus* genus. It is classified inside the Japanese encephalitis serocomplex together with Japanese encephalitis, St. Louis encephalitis, and Murray Valley encephalitis viruses, among others. The *Flavivirus* genus also contains other important pathogens as Dengue, tick-borne encephalitis, or Yellow fever viruses [1], [2], [3]. A wide range of bird species provide the natural hosts for WNV, and the virus cycles between birds and ornithophilic mosquitoes that act as its vector. In addition to birds, WNV also infects multiple vertebrate species including horses and humans. Clinical manifestations of the infection range from asymptomatic or febrile illness to a neuroinvasive disease that can result in fatal encephalitis [4]. WNV has been historically associated with asymptomatic infections and sporadic disease outbreaks in humans and horses in Africa, the Mediterranean basin, the Middle East, and Australia. Nowadays, due to climate warming, changes in vector feeding behaviour, and spread through the globalization of trade and travel, this mosquito-borne flavivirus is worldwide distributed, and has become a global concern [5], [6], [7]. Only in the US, about 3 million infections resulting in about 780,000 illness have been estimated from WNV introduction in 1999 through 2010 [8]. Currently there is no vaccine or specific therapy approved for use in humans [9].

The genome of WNV is a single stranded RNA molecule of positive polarity about 11,000 nucleotides in length [10]. This molecule encodes a single open reading frame that is translated into a single polyprotein. The viral polyprotein is cleaved into three structural proteins –capsid or core (C), pre-membrane/membrane (prM/M) and envelope (E) proteins– and seven non-structural proteins [1], [2], [3]. The genomic RNA is enclosed within a nucleocapsid composed of multiple copies of the C protein, which constitutes the core of the virion and is enveloped by a lipid bilayer [11]. Mature virions are approximately 50 nm in diameter and display a smooth outer surface composed of 180 copies of the small M protein and an equal number of E glycoprotein copies arranged as 90 anti-parallel homodimers resulting in a particle of pseudo-icosahedral symmetry [11].

Virions assemble and bud into the endoplasmic reticulum [12], [13] and require trafficking along the secretory pathway for maturation [13]. During this process, the flavivirus prM protein is cleaved by a cellular furin-like protease within the acidic environment of the *trans*-Golgi network. This orchestrates profound rearrangements of viral glycoproteins that result in the acquisition of the mature structure of the virion [14], [15], [16]. While this cleavage is a required step in the viral life cycle, it can be inefficient; suggesting that complete maturation is not required for infectivity [17], [18]. Indeed, the presence of flaviviral ‘mosaic’ particles combining regions of mature and immature structure has been documented [19], and the degree of maturation of flavivirus particles has been related to different aspects of their interaction with antibodies [18], [20], [21], [22]. An acidic pH step is not only required for viral maturation during WNV infectious cycle, but also during viral entry [23], [24]. The acidic pH inside endosomes triggers rapid conformational rearrangements on the E glycoprotein of internalized virions, leading to the formation of trimers together with the exposure of the hydrophobic fusion loop of the E glycoprotein [23], [25], [26]. These phenomena promote viral membrane fusion and release of the nucleocapsid into the cytosol [23], [24], [25], [26].

Characterization of viruses with altered pH requirements provides both structural and mechanistic information regarding the processes in which acidic pH is involved [27], [28], [29]. A strategy to isolate this class of mutants consists on the selection of mutants resistant to drugs that impair organelle acidification, such as the weak base ammonium chloride, NH_4_Cl [27], [30], [31]. The sensitivity of WNV to treatment with NH_4_Cl has been well documented [16], [32], [33], and the inhibitory effect of this chemical was related to two different processes: the inhibition of endosome acidification during viral entry, hence impairing membrane fusion [32], [33]; and the blockage of furin-mediated cleavage of prM at the *trans*-Golgi, thus impeding virus maturation [16], [18]. In this study, we have isolated and characterized a WNV mutant with increased resistance to the acidotropic agent NH_4_Cl. In contrast to other previously described flaviviruses with altered pH requirements, which exhibited mutations on the proteins that conform the external shell of the virion (E and M) -[34] and references therein-, the WNV mutant here reported displayed a single amino acid substitution on the internal core protein. This amino acid substitution also increased resistance to other acidification inhibitor and induced a reduction of the neurovirulence in mice. The findings here reported unveil a non-previously assessed connection between the viral C protein and the acidic pH involved on entry and maturation of flaviviruses.

## Methods

### Ethics Statement

All animals were handled in strict accordance with the guidelines of the European Community 86/609/CEE at the biosafety animal facilities of the Centro de Investigación en Sanidad Animal of the Instituto Nacional de Investigación Agraria y Alimentaria (CISA-INIA). The protocols were approved by the Committee on Ethics of Animal Experimentation of INIA (permit number 2012-05).

### Cells, Virus, Infections, and Virus Titrations

Vero and baby hamster kidney (BHK)-21 cells were grown as described [35]. Unless specified, all tissue culture reagents were from Lonza (Verviers, Belgium). The origin and history passage of WNV NY99-flamingo382–99 strain has been previously described [10], [35], [36]. For infections performed in liquid medium, Vero cells were washed twice with EMEM before addition of the viral inoculum, and after 1 h at 37°C, the virus was removed and fresh medium containing 2.5% FBS was added. Viral samples were titrated on Vero cells by standard plaque assay in semisolid agarose medium [35]. Viral stocks were produced in the absence of NH_4_Cl and titrated also in the absence of the drug unless specified.

### Drug Treatments

Inhibition of organelle acidification was performed with NH_4_Cl (Merck) or concanamycin A (Sigma, St Louis, MO) as described previously [30]. Cells were treated for 1 h prior to infection with 25 mM NH_4_Cl or 30 min with concanamycin A (10 or 100 nM). Both drugs were maintained throughout the whole infection time to avoid cellular recovery. In all experiments involving NH_4_Cl, extracellular pH was buffered with 25 mM HEPES at pH 7.5 (Sigma). Inhibition of furin-mediated maturation of WNV was performed with furin inhibitor I, decanoyl-Arg-Val-Lys-Arg-chloromethylketone (Calbiochem, Darmstadt, Germany) as reported [21], [22]. Cells were treated 30 min prior to infection with 25 µM furin inhibitor I and the drug was maintained throughout the whole infection time. As the half-life of furin inhibitor I in aqueous solution is about 4 to 8 h [37], additional furin inhibitor I was added at 16 h.p.i. to ensure blockage of the progeny virus maturation [22]. Control cells were treated with the same amount of drug vehicle (H_2_O for NH_4_Cl, and dimethyl sulfoxide, DMSO, for concanamycin and furin inhibitor I).

### Purification of Virions

Supernatants from infected cultures treated or not with NH_4_Cl were harvested 24 h.p.i. and cleared from cellular debris by centrifugation at 850×*g* for 15 min, followed by centrifugation at 12,000×*g* for 30 min. Viral particles were concentrated from cleared supernatants by ultracentrifugation at 141,000×*g* for 2.5 h through a 20% sucrose cushion in PBS. The pellets containing viral particles were resuspended in PBS and analyzed by western blot.

### Immunofluorescence

Immunofluorescence detection of WNV-infected cells was performed following a previously reported protocol [38]. For this purpose, monoclonal antibody 3.67G (Millipore, Temecula, CA) directed against the E glycoprotein of WNV was used in combination with appropriated secondary antibodies labelled with Alexa Fluor 488 (Molecular Probes, Eugene, O).

### Western Blot

WNV glycoproteins from concentrated virions were detected by western blot using monoclonal antibody 3.67G to detect E glycoprotein, or a rabbit polyclonal antibody against the M protein (Imgenex, San Diego, CA), as previously described [38]. Proteins were detected by chemiluminiscence using a ChemiDoc™ XRS+ System (Bio-Rad, Hercules, CA). The intensity of protein bands was quantified with ImageLab™ 2.0.1 software (Bio-Rad).

### In vitro Furin Treatment of Immature Viral Particles

Treatment with furin was performed following a previously described procedure with minor modifications [14], [39]. Briefly, WT and Res virus were purified as described above and dialyzed against PBS to remove the remaining sucrose. Viral samples were mixed with the same amount of 50 mM 4-morpholineethanesulfonic acid, MES (Sigma). Samples were treated or not (control) with furin (New England Biolabs, Ipswich, MA) at 30°C for 16 h in the presence of 3 mM CaCl_2_ and then neutralized with a buffer containing 100 mM Tris at pH 8.0 and 120 mM NaCl. The infectivity in each sample was determined by standard plaque assay in semisolid agarose medium [35]. The number of PFU developed for each virus subjected to furin treatment was counted and expressed as the percentage of infectivity compared to that obtained in untreated samples (considered as 100% of infectivity).

### Acid Sensitivity Assays

WNV acid sensitivity was determined by a modification of the previously described procedure [31], [35]. Aliquots containing about 10^7^ PFU of each virus tested were treated with different amounts of 1 M MES to give a final pH of 7, 6,5 or 6 and incubated 15 min at 37°C. Then, samples were neutralized with the appropriate amount of 5 M NaOH. The pH of the samples was verified after acidification and also after neutralization. Samples prepared in this way were serially diluted and added to Vero cells grown on 96-well plates as for standard TCID_50_ assays. Cells were fixed with 4% formaldehyde and stained with crystal violet one week p.i. [35]. The remaining infectivity in each sample treated with acidic pH was calculated as the reciprocal of the last virus dilution that induced cytophatic effect and expressed as the percentage of infectivity relative to the infectivity in control samples (pH 7, 100% infectivity).

### Analysis of the Genetic Stability of the Mutants

Viruses were subjected to ten serial passages on Vero cells in the absence of NH_4_Cl and sequenced by automatic DNA sequencing (Macrogen Inc., Seoul, Korea) [35]. Biological clones of each virus that had been serially passaged 10 times in the absence of NH_4_Cl were plaque purified and directly sequenced. Sequences were confirmed by at least two independent sequence reactions.

### Viral RNA Extraction, cDNA Synthesis, and DNA Sequencing

Viral RNA was extracted from the supernatants of infected cultures, or from homogenate brain extracts from infected mice [36], [40] using a NucleoSpin Viral RNA Isolation kit (Macherey-Nagel GmbH & Co., Düren, Germany). cDNA was synthesized by reverse transcription of viral RNA using SuperScript™ One-Step RT-PCR with Platinum® Taq (Invitrogen) and the appropriate oligonucleotide primers (sequences provided upon request). The size and purity of the amplified PCR products were checked by agarose gel electrophoresis and ethidium bromide staining. PCR products were purified using a Speedtools PCR Clean-up kit (Biotools B&M Labs S.A., Madrid, Spain), quantified by UV spectrophotometry using Nanodrop equipment (NanoDrop Tecnologies, Wilmington, DE) and sequenced as described above. Nucleotide positions correspond to those described previously for WNV strain NY99-flamingo 382–99 [10].

### Quantitative RT-PCR

Viral RNA was extracted as described above, and the amount of viral RNA copies was determined by quantitative RT-PCR [41] as genomic equivalents to PFU/ml by comparison with RNA extracted from previously titrated samples [36], [40].

### Infectious Clone Manipulation, *in vitro* Transcription of Viral RNA, and Transfection of Eukaryotic Cells

Plasmid pFLWNV [42], which contains the full length cDNA of WNV strain NY99, was used to construct plasmid pFLWNV-A103G bearing the nucleotide substitution found in the NH_4_Cl-resistant WNV reported here. To this end, the DNA fragment (3652 base pairs) encompassed between *Bam*HI and *Sph*I restriction sites of pFLWNV was synthesized *in vitro* containing the nucleotide replacement A103G on the cDNA of WNV by GenScript (Piscataway, NJ). This DNA was cloned between the same restriction sites of CopyControl™ pCC1BAC™ plasmid, and amplified using TransforMax™ EPI300™ *E. coli* competent cells (Epicentre Biotechnologies, Madison, WI). The plasmid was digested using the same restriction endonucleases (Roche, Manheim, Germany) and the insert bearing WNV nucleotide substitution A103G was ligated into pFLWNV digested with the same enzymes using T4 DNA ligase (Roche). ElectroMAX™ DH5α-E™ competent *E. coli* cells (Invitrogen) were transformed with infectious cDNA clones by electroporation using a GenePulser apparatus (Bio-Rad). Nucleotide sequences of plasmids pFLWNV and pFLWNV-A103G were verified by nucleotide sequencing.

Plasmid DNA from infectious clones was purified from bacteria through PureLink™ HiPure Plasmid Filter Maxiprep kit (Invitrogen) and linearized by digestion with *Xba*I (Roche). Viral RNA was synthesized by *in vitro* transcription using the mMESSAGE mMACHINE® kit (Ambion, Austin, TX) as previously described [42]. Viral RNA was transfected into BHK-21 cells using Lipofectin® Transfection Reagent (Invitrogen) as indicated by the manufacturer. Three days post-transfection, the recovered viruses were harvested and titrated on Vero cells. The identity of viral RNA extracted from recovered viruses was also confirmed by nucleotide sequencing.

### Mice Experiments

Mouse experimentation was carried out in our BSL-3 containment facilities. Groups of eight-week-old *Swiss* female mice were intraperitoneally (i.p.) infected with 10^2^, 10^4^ or 10^7^ PFU/mouse of each virus analyzed resuspended in 100 µl of PBS. Mice were kept with *ad libitum* access to food and water during the experiment and were monitored daily for signs of illness up to 15 days p.i. Those mice showing clear signs of disease, as ruffled fur, hunching, hind limb weakness, and paralysis, were anesthetized and sacrificed, as were all surviving mice at the end of the experiments.

### Data Analysis

To test the significance of the differences, analysis of the variance (ANOVA) was performed with statistical package SPSS 15 (SPSS Inc, Chicago IL), applying Bonferroni’s correction for multiple comparisons. Data are presented as mean ± standard deviation (SD). Asterisks (*) in the figures denote statistically significant differences (*P*<0.05). Kaplan-Meier survival curves were analyzed by a logrank test using the statistical package GraphPad PRISM v.2.01 (GraphPad Software). The median survival time (MST) was calculated for every group of inoculated mice.

## Results

### Selection of a WNV Population with Increased Resistance to NH_4_Cl

WNV with increased resistance to NH_4_Cl was selected from a cell culture passaged WNV strain [36] responsible for the outbreak of encephalitis in NY in 1999 (NY99) [10], here termed WT. To this end, WNV virus was propagated on Vero cells in the presence of 25 mM NH_4_Cl, until cytophatic effect was clearly visible, about 3 days postinfection (p.i.). At this time point, the virus progeny was harvested and used to infect fresh Vero cells in the presence of NH_4_Cl. Serial infections were repeated 10 times in the presence or in the absence of the drug (control conditions). Viruses from passage 10 were amplified by a subsequent round of infection in absence of NH_4_Cl and used in further experiments, thus preventing possible bias derived from the production of immature particles due to NH_4_Cl treatment.

**Figure 1:**
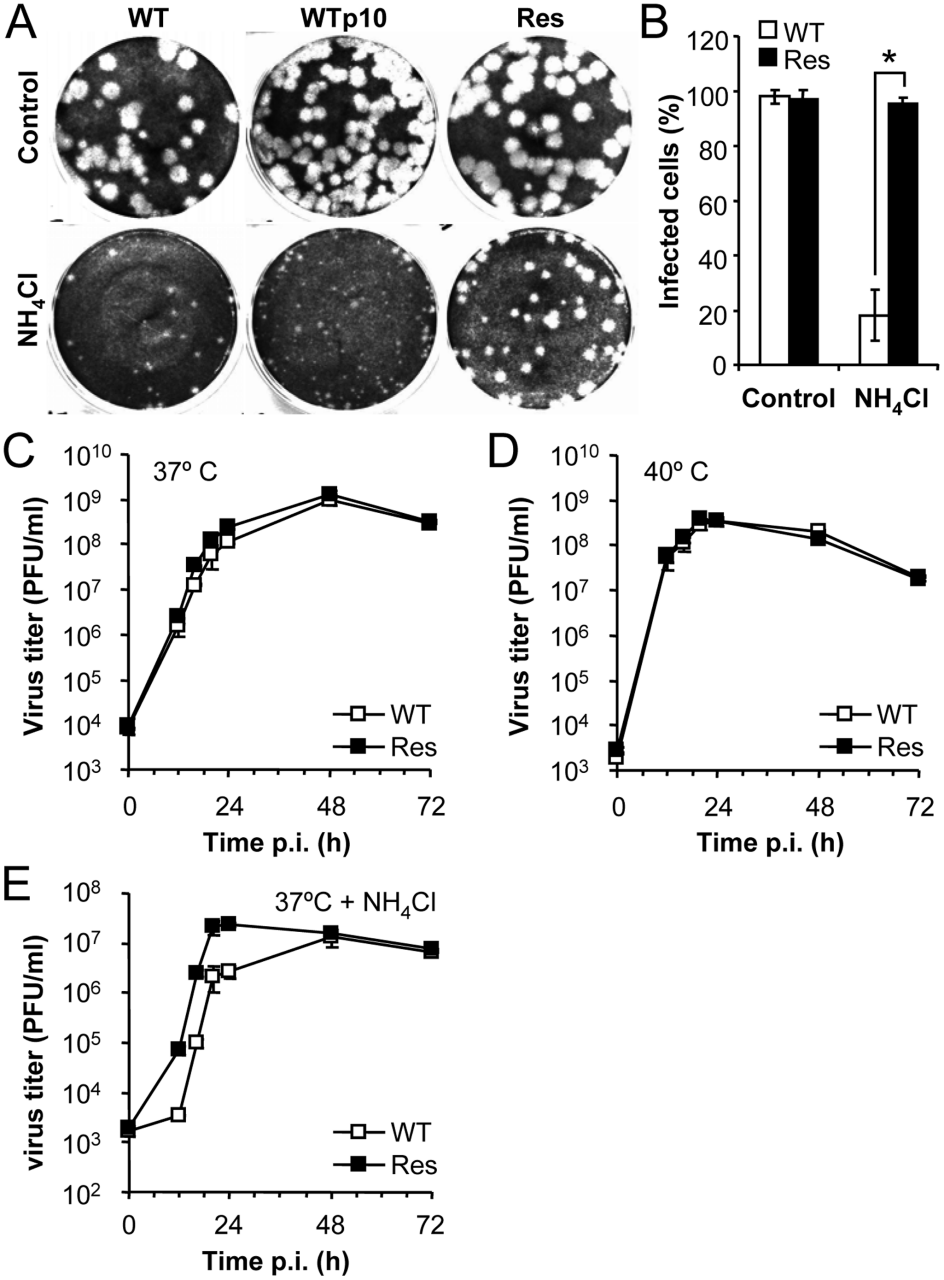
Isolation and growth properties of a WNV population with increased resistance to NH_4_Cl. (**A**) Lysis plaques produced by different populations of WNV in the absence (Control) or in the presence of 25 mM NH_4_Cl. WT corresponds to WNV NY99. WTp10 correspond to a population of WNV WT selected after 10 passages without NH_4_Cl. Res corresponds to a population of WNV WT selected after 10 passages in the presence of NH_4_Cl. Lysis plaques were visualized by staining of Vero cells with crystal violet 3 days p.i. (**B**) Vero cells treated or not with 25 mM NH_4_Cl were infected with WT or Res WNV (MOI of 0.01 PFU/cell). The percentage of infected cells (positively stained with an antibody against the E glycoprotein) was determined by immunofluorescence and confocal microscopy at 48 h.p.i. (**C**) Time-course analysis of infectious progeny release by cells infected with WT or Res WNVs. Vero cells were infected (MOI of 0.5 PFU/cell) at 37°C and the virus progeny released to the medium was determined by plaque assay at different times p.i. (**D**) Cells infected as in (C) were incubated at 40°C and the viral titers in the supernatants were determined as described [35]. (**E**) Cells infected as in (C) were incubated at 37°C in the presence of 25 mM NH_4_Cl, and the viral titer in supernatant was determined. Error bars represent SD. The data presented are product of three independent experiments.

The degree of resistance to NH_4_Cl was assayed on these viral populations by plaque assay performed in the presence or in the absence of NH_4_Cl (Fig. 1A). In the presence of NH_4_Cl, the diameter of the lysis plaques developed by the WT virus at 3 days p.i. (1.10±0.27 mm) was smaller (44% reduction, *P*<0.001) than that produced by the population passaged 10 times in the presence of NH_4_Cl (1.97±0.53 mm), hence named Res (abbreviation of Resistant) WNV. However, both viruses displayed similar plaque sizes (WT, 3.34±0.76 mm; Res, 3.33±0.80) in absence of NH_4_Cl. To rule out that this difference between WT and Res WNVs was derived from tissue culture adaptation upon serial passages, a population of WNV WT passaged 10 times in absence of NH_4_Cl (termed WTp10) was included in the analysis. This viral population showed no increase in plaque size compared to that of WT when plated in the presence of NH_4_Cl (0.98±0.29 mm), supporting the specificity of the increase in plaque size noted in the NH_4_Cl Res population. The sensitivity to NH_4_Cl of Res population was further analyzed by immunofluorescence staining of infected cells (Fig. 1B). No significant differences were noted between cells infected with WT or Res WNVs when infections were performed in the absence of NH_4_Cl, but in cells treated with NH_4_Cl, a statistically significant reduction (about 80%, *P*<0.001) of the percentage of infected cells was observed for WT virus when compared to Res WNV.

As acidic pH is a key for distinct steps of the flavivirus infectious cycle (see Introduction), alterations responsible for the increase in NH_4_Cl resistance of Res virus could carry a reduction of viral fitness. To test this hypothesis, the growth kinetics of both WT and Res WNVs were compared by titration of viruses released to the extracellular medium at 37 or 40°C (Fig. 1 C and D). Both viruses shared similar growth curves at the two temperatures analyzed. However, when time course analysis of the virus released to the extracellular medium was analyzed in the presence of NH_4_Cl, an increase of about one order of magnitude was noticed for Res virus during the first 24 h of infection (Fig. 1E), confirming the partial resistance to NH_4_Cl of Res population. Overall, these results indicate that Res population has an increase in the resistance to NH_4_Cl treatment which carries no major alterations of viral fitness under normal culture conditions.

### Resistance to NH_4_Cl also Confers Resistance to Other Inhibitor of Acidification

**Figure 2:**
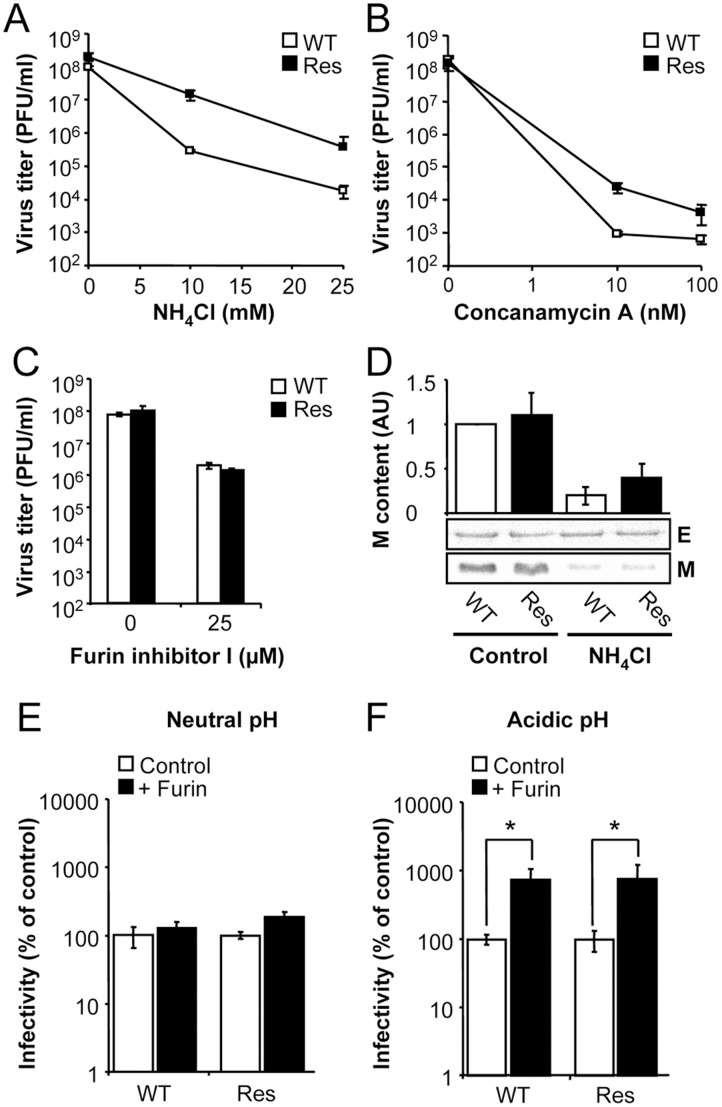
Resistance to NH_4_Cl also increases the resistance to other inhibitor of acidification. Vero cells treated with NH_4_Cl (**A**), concanamycin A (**B**) or furin inhibitor I (**C**) were infected with WT or Res WNVs (MOI of 0.5 PFU/cell). Virus yield was determined by plaque assay at 24 h.p.i. as described [35]. (**D**) Cells treated or not with NH_4_Cl were infected with WT or Res WNVs. Viral particles released to the infectious medium were concentrated by ultracentrifugation through a 20% sucrose cushion. The content of the E and M proteins was determined by western blot as described in Material and Methods. The graph displays the M content relative to the E content in each sample. AU, arbitrary units. (**E** and **F**) Effect of *in vitro* furin treatment on the infectivity of WT and Res virus. WT or Res virus grown in the presence of NH_4_Cl was purified and treated in vitro with recombinant furin at neutral (**E**) or acid pH (**F**). See Methods for details. The remaining infectivity in each sample was determined by standard plaque assay and expressed as percentage of infectivity compared to each control sample untreated with furin (considered as 100% of infectivity). Error bars represent SD. The data presented are product of three independent experiments.

The sensitivity to NH_4_Cl of Res virus was compared to that of WT by performing infections in liquid medium and determining the virus yield at 24 h.p.i. (Fig. 2A). WT and Res WNVs displayed dose-response behaviour against increasing concentrations of NH_4_Cl. Virus yield of Res WNV was about one order of magnitude higher than that of WT in cultures treated with either 10 or 25 mM NH_4_Cl, confirming the selective advantage of this population against treatment with NH_4_Cl.

Since the effect of NH_4_Cl treatment is the inhibition of acidification, the effect of concanamycin A, another inhibitor of acidification [43], on the infection WT and Res WNVs was also analyzed (Fig. 2B). Treatment with concanamycin A resulted in a decrease of virus yield of both viruses, although virus yield of Res WNV was again about one order of magnitude higher than that of WT virus in cells treated with either 10 or 100 nM concanamycin A. This indicates that resistance to inhibition of acidification induced by treatment with NH_4_Cl also confers resistance to another drug as concanamycin A, suggesting that the viral mechanism of resistance to acidification inhibitors is common for both inhibitors.

Acidic pH has been related to furin-mediated cleavage of flavivirus prM during virus maturation, and also related to viral particle infectivity (see Introduction). Along this way, we studied if the mechanism of resistance to inhibition of organelle acidification also conferred resistance to inhibition of furin-mediated maturation of viral particles. To this end, the effect of furin inhibitor I (decanoyl-Arg-Val-Lys-Arg-chloromethylketone), which has been reported to specifically inhibit this cleavage in WNV and other flaviviruses as Dengue virus [21], [22], was assayed on the infection of WT and Res WNVs (Fig. 2C). A similar reduction of the virus titer (compared to untreated cells) of both WT and Res WNVs was observed, thus showing that infection with Res virus was not resistant to inhibition of furin-mediated maturation of viral particles. To further study possible alterations on maturation, the degree of maturation of WT and Res viral particles was also investigated (Fig. 2D). Viral particles produced in the presence or in the absence of NH_4_Cl were concentrated by ultracentrifugation and the amount of mature M protein was analyzed by western blot. These experiments showed that viral particles produced in the presence or in the absence of NH_4_Cl of WT and Res WNVs displayed a similar reduction in the amount of mature M protein, suggesting that furin-mediated cleavage of prM was impaired by NH_4_Cl for both WT and Res virus.

The furin cleavage site in prM is only exposed when the virus has undergone maturation [14], [39]. In this way, treatment of flavivirus immature particles with furin at acidic pH increases the infectivity of these particles by induction of the pH-induced rearrangements that allow furin-mediated cleavage of prM [39]. To test the accessibility of furin cleavage site at neutral and low pH, both WT and Res virus produced in the presence of NH_4_Cl (to prevent acid-induced rearrangements and furin cleavage at the *trans*-Golgi) were treated or not with furin at neutral or acidic pH and the infectivity in each sample was determined (Fig. 2E and F). No significant increase in virus infectivity was observed for WT or Res virus treated with furin at neutral pH, when compared to untreated samples (Fig. 2E). However a significant increase in the infectivity of viral samples treated with furin at acid pH was noticed, and the extent of this increase was similar for WT and Res virus (Fig. 2F). This suggests that the structural change necessary to expose the furin cleavage site in Res virus requires acidic pH, as observed for WT. Overall, these results confirm that the mechanism of resistance to inhibition of acidification of Res WNV is not related to alterations in viral particle maturation.

### Relationship between Viral Genome Copies and Infectivity

The production of viral RNA in infected cultures treated with NH_4_Cl, concanamycin A or furin inhibitor I was analyzed by quantitative RT-PCR and determined as genomic equivalent to PFU/ml by comparison with previously titrated samples. These values were compared to the content on infectious particles (PFU/ml), and the PFU/genomic equivalent ratio was calculated as an indicator of the specific infectivity per viral genome (Table 1). Res WNV displayed an increase of about one order of magnitude in the ratio PFU/genomic equivalent when compared to WT virus upon NH_4_Cl, or concanamycin A, treatment. However, similar PFU/genomic equivalent ratio was obtained for both WT and Res viruses in control cells or in those treated with furin inhibitor I (Table 1). In this way, WT and Res WNVs produced similar amounts of infectious particles when furin-mediated maturation was impaired (cells treated with furin inhibitor I), but Res virus produced about ten-fold more infectious particles than WT upon treatment with inhibitors of acidification.

**Table 1:** Relationship between infectivity and genomic RNA copies.

	Virus	Treatment			
		Control	NH_4_Cl	Concanamycin A	Furin inhibitor I
Infectivity (PFU/ml)	WT	9.7×10^7^±1.3×10^7^	2.9×10^5^±1.0×10^4^	9.7×10^2^±1.2×10^2^	3.2×10^6^±2.9×10^4^
	Res	2.1×10^8^±5.2×10^7^	1.5×10^7^±4.8×10^6^	2.5×10^4^±9.1×10^3^	1.8×10^6^±3.1×10^5^
RNA copies (Genomic equivalent to PFU/ml)*^d^*	WT	1.0×10^8^±4.2×10^7^	8.9×10^6^±4.2×10^4^	6.3×10^6^±5.9×10^5^	1.8×10^8^±4.7×10^7^
	Res	1.7×10^8^±3.3×10^7^	3.1×10^7^±8.0×10^6^	1.9×10^7^±5.7×10^6^	1.2×10^8^±4.2×10^7^
Infectivity/RNA copies (PFU/genomic equvalent)	WT	1.0±0.5	0.033±0.0008	0.00015±0.0002	0.18±0.01
	Res	1.3±0.2	0.47±0.3	0.0013±0.003	0.15±0.05

Data are sumarized as mean ± SD (3 to 6 determinations). Drug treatments used: 10 mM NH_4_Cl, 10 nM Concanamycin A, or 25 µM furin inhibitor. Control indicates that no drug was added to the culture medium. The PFUs in the sample were determined by standard titration [35] at 24 h.p.i. The amount of genomic RNA copies was measured by quantitative RT-PCR (24 h. p.i.) using previously titrated samples, and expressed as genomic equivalents to PFU/ml [40], [41].

### Acid-inactivation Analyses

**Figure 3:**
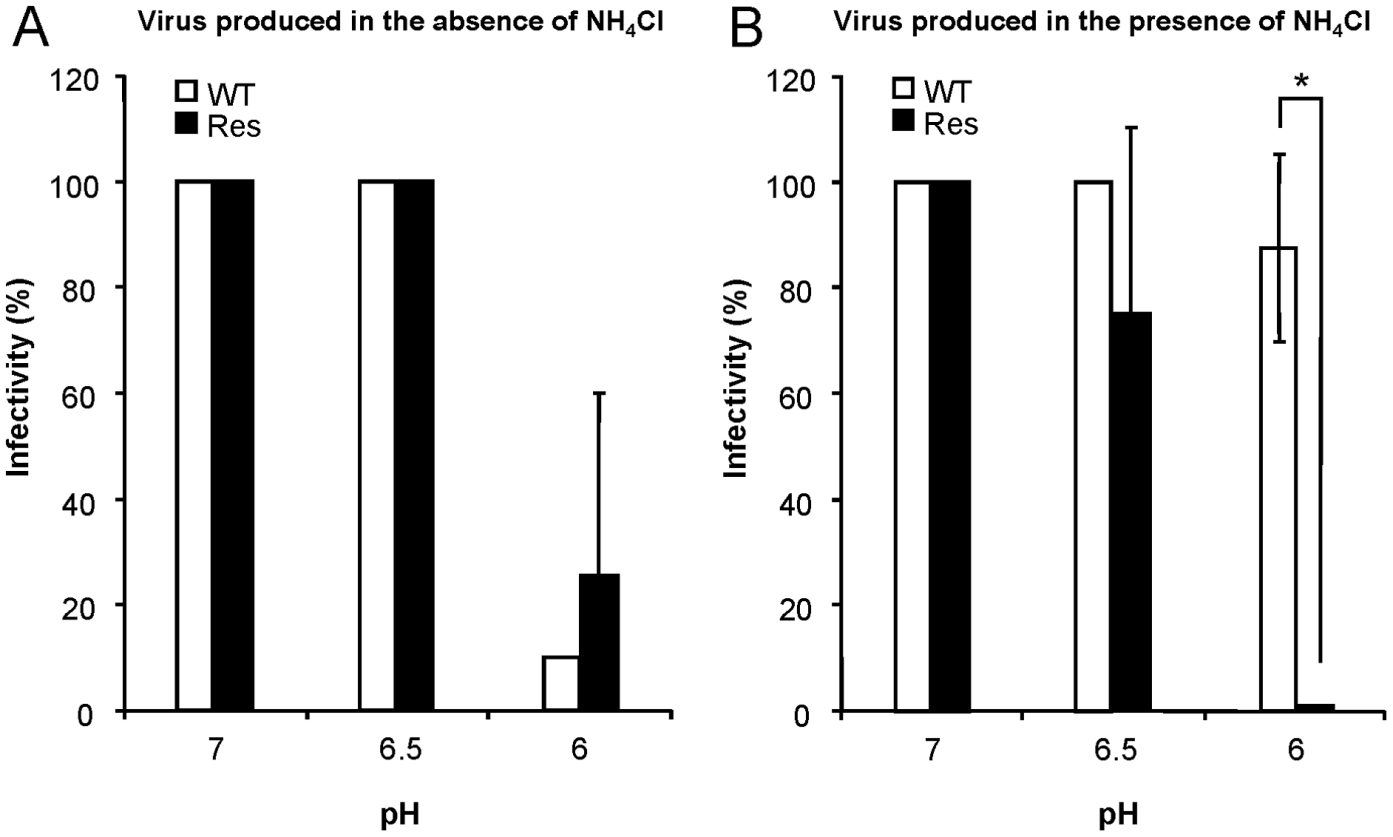
Acid inactivation analysis of the WNV population with increased resistance to NH_4_Cl. Acid-inactivation was analyzed on viruses produced in the absence (**A**) or in the presence of NH_4_Cl (**B**). Equal titers of the WT and Res viruses were incubated at different pHs, neutralized and plated as described in Material and Methods. Infectivity was calculated as the percentage of PFU recovered in relation to that obtained at pH 7. Error bars represent SD. The data presented are product of two independent experiments.

As commented in the Introduction, WNV fusion is triggered by acidic pH inside endosomes. According to this mechanism, the exposure of WNV to acid media in the absence of target membranes results in E protein rearrangement, premature exposure of the fusion loop, and rapid and irreversible inactivation of fusion competence causing a loss of infectivity [32], [44]. Analysis of the acid-induced inactivation of both WT and Res virus showed that, in the case of viruses produced in the absence of NH_4_Cl, which mainly consist on mature viral particles, no significant differences were found between WT and Res virus (Fig. 3A). Similar assays were also performed using viral preparations produced in the presence of NH_4_Cl (immature particles) (Fig. 3B). While no significant differences were observed between both viruses at pHs 7 or 6.5, the infectivity of Res virus produced in the presence of NH_4_Cl was significantly reduced at pH 6 when compared to that of WT virus produced in the same conditions, and also evidenced that WT virus grown on NH_4_Cl was not significantly inactivated upon exposure to acidic pH across the pH range analyzed. These results point that mature particles of Res virus do not display major alterations of fusion pH threshold when compared to WT virus, whereas Res particles produced in the presence of NH_4_Cl can be inactivated by acidic pH in a similar way than those produced in the absence of NH_4_Cl (in contrast to WT virus). Overall these experiments suggest that Res WNV is able to produce more fusion-competent particles (infectious particles) in the presence of NH_4_Cl than WT.

### Genetic Determinants of the Resistance to NH_4_Cl

**Figure 4:**
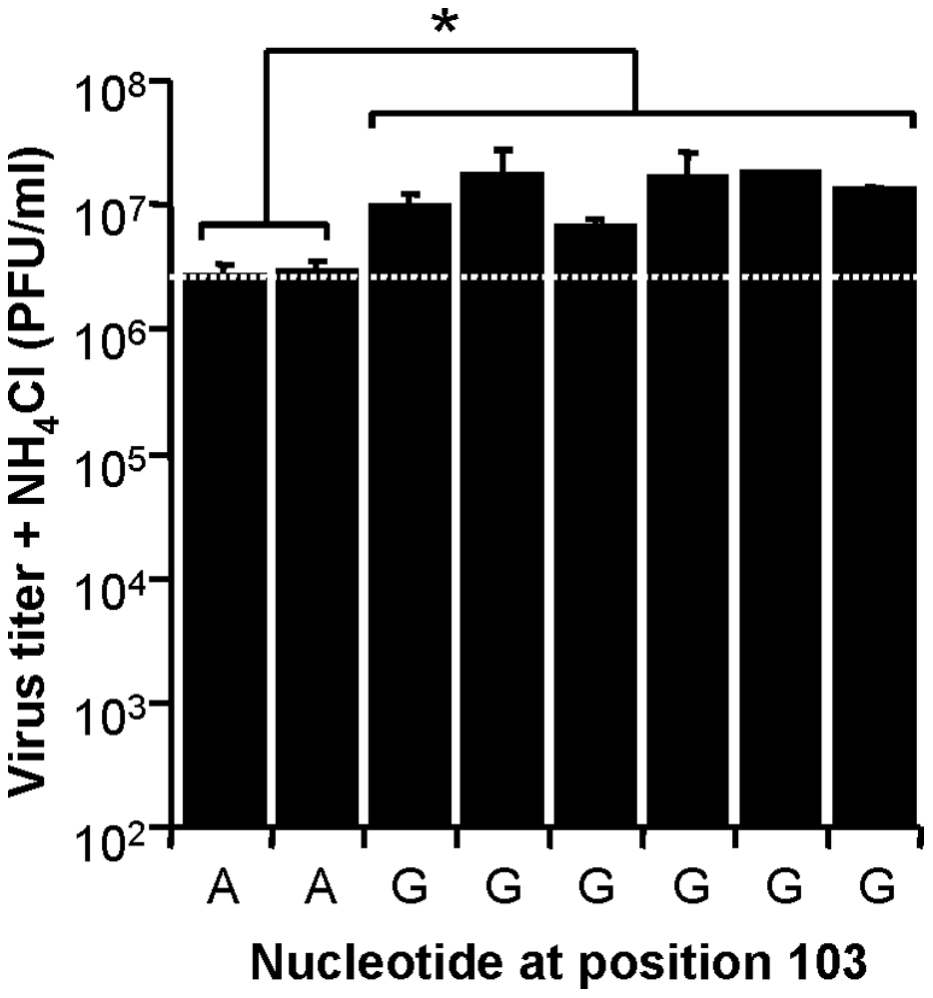
Nucleotide replacement A103G enhances production of infectious particles in the presence of NH_4_Cl. Different biological clones were isolated from Res population and used to infect Vero cells in the presence of NH_4_Cl (MOI of 0.5 PFU/cell). Virus titers were determined 24 h.p.i. as described [35]. The nucleotide at position 103 is indicated for each individual clone. Dashed white line indicates the virus titer shown by biological clones bearing the same nucleotide at this position (A103) than the WT. Error bars represent SD. The data presented are product of three independent experiments.

The genetic basis of resistance to inhibitors of acidification was analyzed in the Res WNV. The complete genomic sequences of the parental WT and Res populations were determined and compared (Table 2). Seven synonymous changes (T4107C, A5280G, T7015C, C8235T, T8811C, T10338C and A10851G) were found in the consensus sequence of both viruses when compared with the reference parental sequence WNV strain NY99-flamingo 382–99 (GenBank accession no. AF196835.2) [10]. The presence of these silent replacements has been previously reported for the WT population, as a probable reflect of the passage history of the virus [35]. In addition, a single non-synonymous nucleotide change was found imposed in the Res population. This non-synonymous replacement, the transition A103G, was responsible for the amino acid replacement K3E in the C protein. Two additional positions in the Res population displayed non-synonymous transitions (G7183A and A7184G), located on the coding region of NS4B protein, although these substitutions were not dominant in the population. The selection of A103G nucleotide substitution was traced along passage history of Res population. This nucleotide substitution appeared at a detectable level at passage 4 and increased with passage number. Selection of this nucleotide substitution was not observed for the viral population that was propagated in absence of NH_4_Cl (data not shown). Eight biological clones were isolated from Res population by amplification of individual lysis plaques and the virus yield produced by these clones in the presence of NH_4_Cl was analyzed. Mean virus titer of the clones (six) that carried nucleotide substitution A103G was significantly higher (*P*<0.021) than those of the clones (two) with WT nucleotide at this position (Fig. 4). Taken together, these results support that the nucleotide replacement A103G, which introduces amino acid replacement K3E in the WNV C protein, is responsible for the increase of resistance to treatment with NH_4_Cl exhibited by Res WNV.

**Table 2:** Nucleotide substitutions found in the population of the WNV resistant to NH_4_Cl.

Genomic region	Nucleotide replacement	
	WT	Res
5′ NCR		
C		A103G (**K3E**)
prM		
M		
E		
NS1		
NS2A	T4107C	T4107C
NS2B		
NS3	A5280G	A5280G
NS4A		
NS4B	T7015C	T7015C
		G7183A (**D90V**) Mixture
		A7184G (**D90G**) Mixture
NS5	C8235T	C8235T
	T8811C	T8811C
	T10338C	T10338C
3′ NCR	A10851G	A10851G

Nucleotide replacements found on the genomic sequence of WT and Res WNVs were compared to WNV strain NY99-flamingo 382–99 (GenBank accession no. AF196835.2) [10]. Amino acid replacements are shown in bold within parentheses. Mixture indicates that this substitution could be detected on the chromatogram although it was not dominant in the population.

### The Single Amino Acid Substitution K3E in the WNV C Protein is Sufficient to Increase Resistance to Inhibition of Acidification

**Figure 5:**
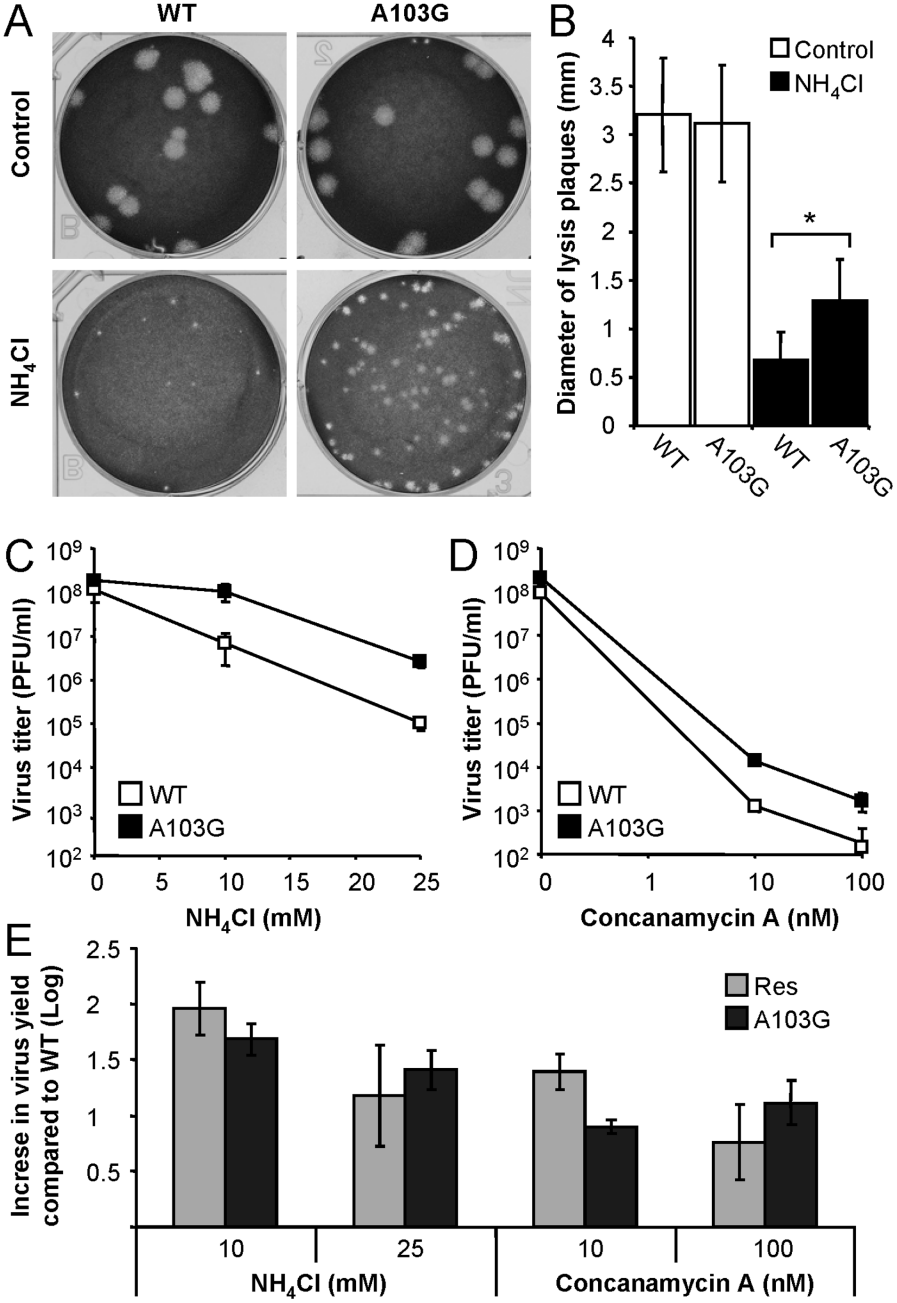
The nucleotide replacement A103G is sufficient to increase resistance to inhibitors of acidification. (**A**) Lysis plaques produced by WT and A103G WNVs recovered from infectious cDNA clones pFLWNV and pFLWNV-A103G, respectively. Viruses were plated in the absence (Control) or in the presence of 25 mM NH_4_Cl and lysis plaques were visualized by staining of Vero cells with crystal violet 3 days p.i. (**B**) Quantitative analysis of the lysis plaque size produced by the different populations of WNV in (A). Vero cells treated with NH_4_Cl (**C**) or concanamycin A (**D**) were infected (MOI of 0.5 PFU/cell) with WT or A103G viruses recovered from the infectious clones described in (A) and the virus titers were determined by plaque assay at 24 h.p.i. as described [35]. (**E**) Comparison of the degree of resistance between A103G and Res virus. The resistance against inhibition of organelle acidification exerted by NH_4_Cl or Concanamycin A was calculated as the increase in virus yield compared to the virus yield displayed by WT viruses under each condition. Error bars represent SD. The data presented are product of three independent experiments.

The role of nucleotide substitution A103G was further evaluated by introducing this replacement into the infectious clone pFLWNV, which contains the full length cDNA of the WNV strain used in this study [42]. Viral RNA was synthesized from both infectious cDNA clones by *in vitro* transcription and transfected into BHK-21 cells. The resistance to NH_4_Cl of viruses recovered from infectious clones was first analyzed by plaque assay performed in the presence or in the absence of NH_4_Cl (Figs. 5A and B). Both viruses displayed similar plaque size (WT, 3.21±0.59 mm; A103G, 3.12±0.60) in the absence of NH_4_Cl. However, when plated in the presence of NH_4_Cl, the diameter of lysis plaques developed by the WT virus was smaller (0.68±0.29 mm, 47.3% reduction, *P*<0.001) than that produced by the A103G virus in the presence of NH_4_Cl (1.28±0.43 mm). The sensitivity to NH_4_Cl of A103G virus was compared to that of WT by performing infections in liquid medium (Fig. 5C). Although both viruses grew at a similar level in the absence of the drug, virus yield of A103G virus was about one order of magnitude higher than that of WT virus in cultures treated with either 10 or 25 mM NH_4_Cl, confirming that the introduction of this mutation into WNV increased resistance to NH_4_Cl. The sensitivity to concanamycin A was also lower for A103G virus (Fig. 5D). The degree of resistance of A103G virus was similar to that of Res virus (Fig. 5E), thus confirming that the single amino acid substitution K3E in the WNV C protein, derived from introduction of the nucleotide substitution A103G, is sufficient to increase resistance to inhibitors of acidification and completely responsible of the phenotype of Res virus.

### Comparative Genomics of WNV C Protein

**Figure 6:**
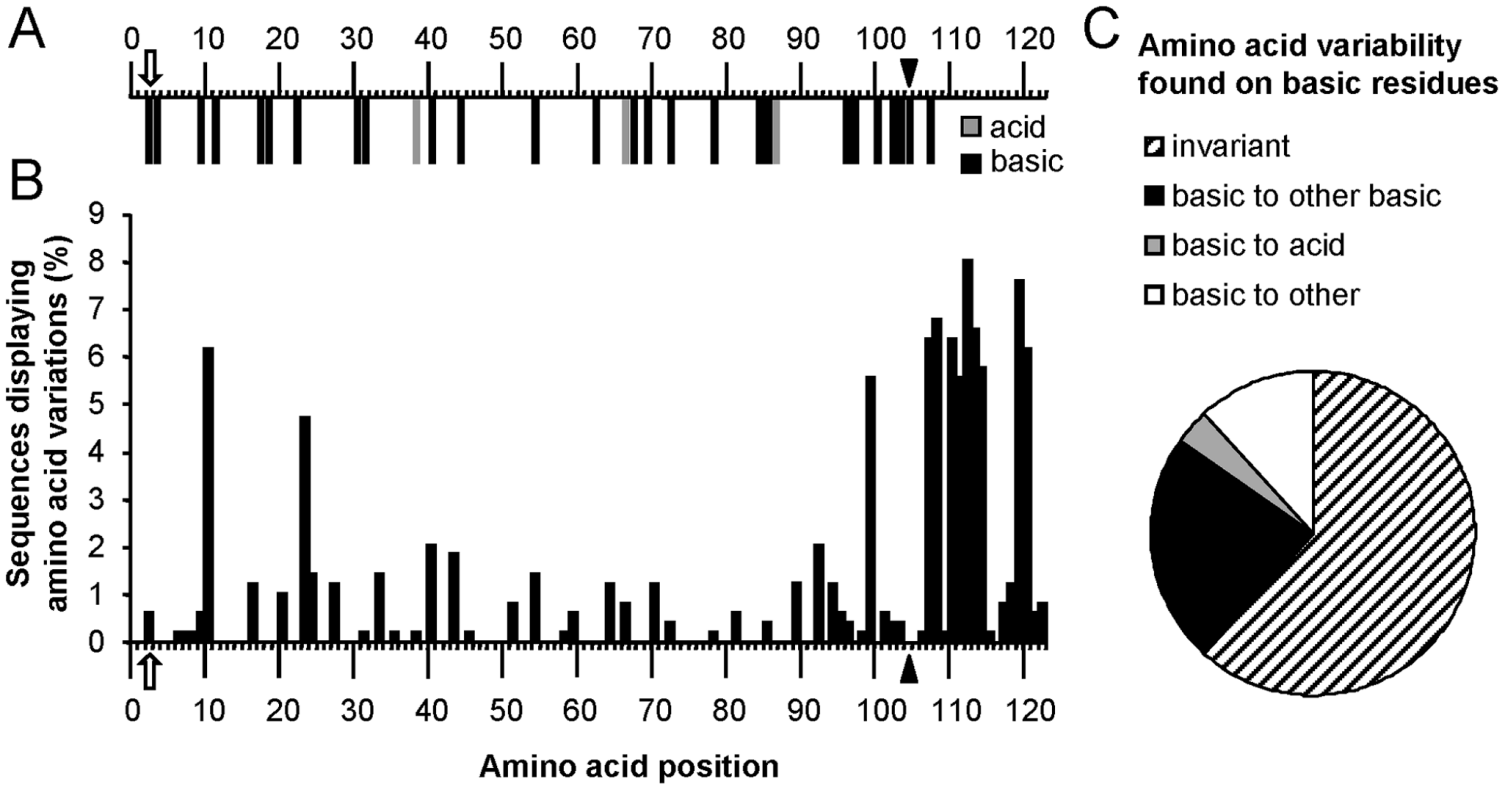
Comparative genomics analysis of the WNV C protein. (**A**) Distribution of basic (K and R) and acid (D and E) residues along the WNV C protein from strain NY99-flamingo 382–99. (**B**) Analysis of the variability of amino acids inside the WNV C protein. A total of 486 sequences from isolates that varied geographically and temporally that are available at GenBank were compared, and the percentage of sequences showing amino acid variation was analyzed for each position. (**C**) Analysis of the amino acid variation of basic residues of the WNV C protein. White arrow points to the position mutated that increased resistance to NH_4_Cl. Black arrowhead indicates the site of maturation cleavage [64].

The amino acid substitution K3E found on Res WNV, located on the N–terminus of C protein -which is presumed to be a disordered region (Fig. S1A and [45])- involves replacement of a basic residue for a negatively charged one. The WNV C protein is a highly basic protein containing 26 basic residues (K or R), and only 3 acid residues, D or E (Fig. 6A). This prompted us to analyze if this type of charge replacement amino acid substitution was frequent by comparing 486 sequences of the WNV C protein from isolates that varied geographically and temporally and are available at GenBank. The criterion for inclusion in the analysis was that the deposited sequence covered at least 98% of the C protein. Sequence variability was similar along the C protein, except for a highly variable region located at the hydrophobic C-terminus, after the maturation cleavage site (Fig. 6B and S1B). Comparison of amino acid sequences revealed that the K3 was invariant in 99.38% of sequences analyzed (Fig. 6B). The remaining 0.62% of the analyzed sequences (3 sequences) corresponded to isolates of the genetically divergent Rabensburg lineage that carried other basic residue (R) in this position (ADG36441.1, ADG36442.1, and AAW81711.1). This analysis highlights the importance of the conservation of a positive amino acid residue in this location. Even more, given the 26 basic residues of WNV C protein, 16 were found to be invariant, 6 tolerated permutation for other basic residue, only 1 was replaced by an acid residue (K10D in ADG36441.1, ADG36442.1, and AAW81711.1), and 3 tolerated variation to other non-basic, non-acid residues (Fig. 6C). These analyses also revealed that substitutions leading to the introduction of negatively charged residues were very infrequent. Within the sequences analysed, only 6 (1.2%) showed this kind of variations: G7E (ADK62441.1), G9D (ADK62428.1), and the previously mentioned K10D from Rabensburg lineage (ADG36441.1, ADG36442.1, AAW81711.1). Considering the potential relevance of the presence of basic residues on the N-terminus of the flaviviral C proteins, the analysis was also extended to the C protein of other flaviviruses, unveiling that K3 together with K4 were conserved among several flaviviruses of the Japanese Encephalitis serocomplex, and that other members of the flavivirus genus (*i.e.* Dengue and Yellow Fever viruses) also presented accumulation of basic residues at the N-terminus of the protein (Fig. S2). Overall, these findings reinforce the functional importance of positively charged amino acids on the N-terminus of the WNV C protein.

### NH_4_Cl-resistant WNV Displays Reduced Virulence *in vivo*

**Figure 7:**
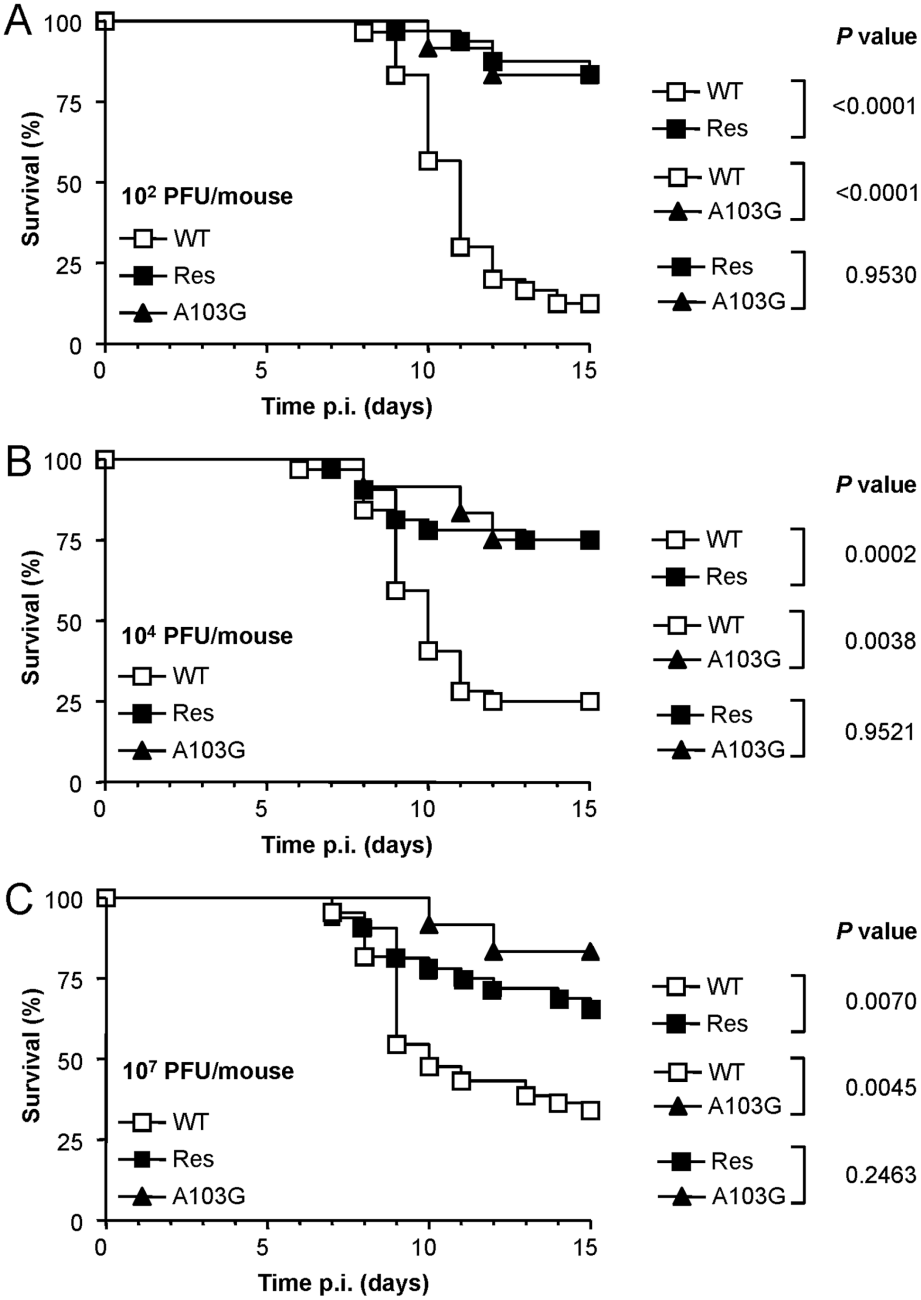
Mice survival after inoculation with NH_4_Cl-resistant WNV. Groups of eight-weeks old mice were i.p. infected with 10^2^ (**A**), 10^4^ (**B**), or 10^7^ (**C**) PFU/mouse of WT, Res, and A103G WNVs, and monitored daily for 15 days. WT and Res virus correspond to data pooled of three independent experiments with similar results (32 to 44 animals per dose). Values of A103G virus correspond to a single experiment (12 animals per dose). The *P* values for specific comparisons of mortality are shown.

The *in vivo* infectivity of NH_4_Cl-resistant viruses was analyzed using a murine infection model for WNV. Mice were i.p. infected with different doses (10^2^, 10^4^, or 10^7^ PFU/mouse) of WT, Res and A103G viruses and the mortality was recorded along time (Fig. 7A, B and C). Animals inoculated with Res or A103G viruses displayed a significant reduction of the mortality when compared to the WT at the three doses assayed, whereas the mortality induced by Res and A103G was not significantly different. Median survival times (MSTs) for mice inoculated with WT virus ranged from 10 (10^7^ PFU/mice) to 11 days (10^2^ PFU/mice), while those of mutant viruses were >15 days. A lack of dose response in the survival rates for animals infected with WT or A103G virus was noticed, and survival curves of animals infected with Res mutant also displayed a weak dose-response. This is compatible with previous observations of a lack of a direct relationship between WNV infecting dose and mortality rates reported by us and others [36], [46], [47], [48]. As none of the doses assayed for Res or A103G virus killed at least 50% of the animals, the exact determination of 50% lethal dose (LD_50_) for mutant viruses was impaired. Albeit from these results, it could be extrapolated that LD_50_ of A103G and Res virus was >10^7^ PFU/mice, whereas LD_50_ of WT virus was <10^2^ PFU/mice (Fig. 7A), thus showing a difference of more than 5 orders of magnitude between WT and NH_4_Cl-resistant viruses.

### Genetic Stability of Nucleotide Substitution A103G

To analyze the stability of the selected mutation in the absence of NH_4_Cl, Res or A103G viruses were subjected to ten serial passages in the absence of NH_4_Cl and the identity of the genomic region encoding the structural proteins (C, prM/M and E) was determined by nucleotide sequencing (Table 3). The nucleotide substitution A103G was found in the population after 10 serial passages in three out of three experiments performed for Res virus, and similar results were obtained for A103G virus. No additional nucleotide substitution was noticed, except for the silent nucleotide replacement G813A, located in the genomic region that encodes M protein, fixed during the second experiment performed with A103G virus. The frequency of viruses bearing nucleotide substitution A103G in the population was also investigated by analysis of biological clones selected from the population of either Res or A103G viruses obtained after ten passages in the absence of NH_4_Cl (Table 3). All biological clones analyzed carried the nucleotide substitution A103G, except for one experiment performed with Res virus in which 2 clones out of 9 analyzed exhibited a direct reversion to WT. Overall these results indicated that, nucleotide substitution A103G remained dominant in the population after 10 serial passages, although the generation of revertants can be also noticed.

**Table 3:** Genetic stability of A103G nucleotide substitution after ten serial passages in the absence of NH_4_Cl.

	Experiment	Nucleotide substitutions found in the sequence of genomic region encoding WNV structural proteins	Proportion of biological clones bearing nucleotide substitution A103G
**Res**	1	A103G (**K3E**)	8/8 (100%)
	2	A103G (**K3E**)	7/9 (77.8%)
	3	A103G (**K3E**)	N.D.
**A103G**	1	A103G (**K3E**)	8/8 (100%)
	2	A103G (**K3E**), G813A	8/8 (100%)
	3	A103G (**K3E**)	N.D.

Mutant WNVs (Res population or A103G virus recovered from infectious clone pFLWNV-A103G) were subjected to 10 serial passages on Vero cells. Viral RNA was extracted and amplified by RT-PCR and the nucleotide sequence of the genomic region encoding the structural proteins was determined. Nucleotide replacements found on the genomic sequence of either Res or A103G subjected to 10 serial passages in the absence of NH_4_Cl were compared to WNV strain NY99-flamingo 382–99 [10] and to WNV recovered from plasmid pFLWNV [42] respectively. Amino acid replacements are shown in bold within parentheses. The presence of nucleotide substitution A103G was analyzed by nucleotide sequencing on different biological clones from each population subjected to 10 serial passages in the absence of NH_4_Cl. Each biological clone corresponded to an individual lysis plaque produced by infection in semisolid agar medium. N.D. Not done.

**Figure 8:**
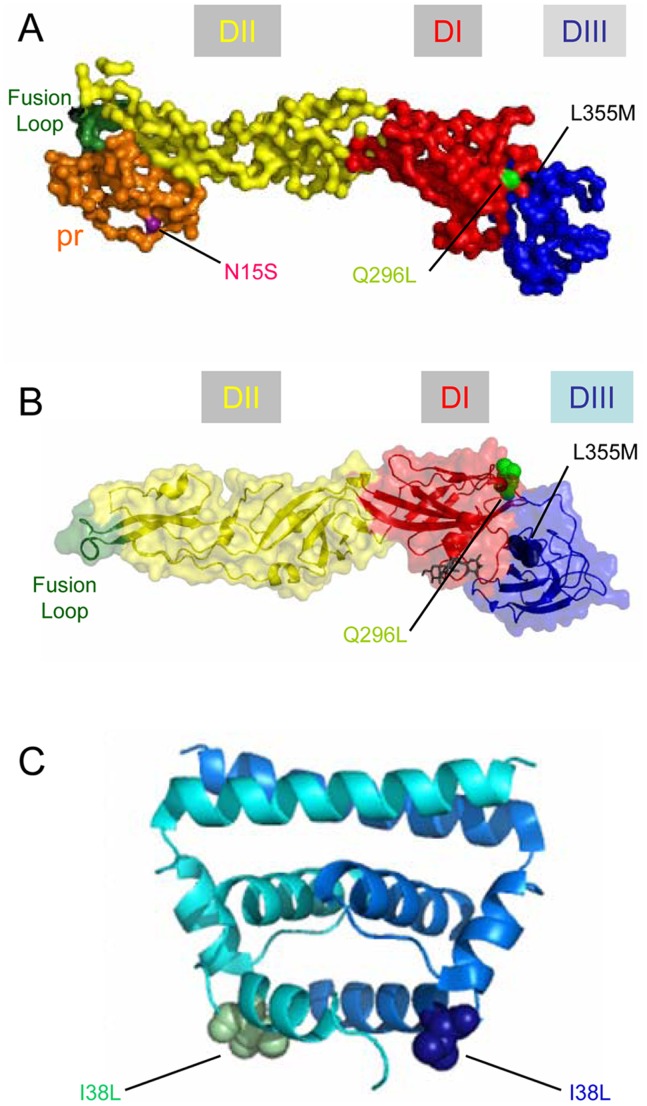
Location of mutated residues found in viruses recovered from mice infected with Res virus. (**A**) Structure of WNV pr/E complex based on cryo-electron microscopy reconstruction [20]. (**B**) Crystal structure of WNV E glycoprotein [82]. The positions of amino acid replacements Q296L (light green) and L355M (black) found in the E glycoprotein of virus N.2.1 and amino acid replacement N15S (purple) found in the pr peptide (orange) of virus N.4.3 are highlighted. Color code for E glycoprotein: DI, red; DII, yellow; DIII, blue; fusion loop, green. (**C**) Ribbon diagram showing the location of I38L amino acid substitution found in virus N.4.1 in the structure of the central portion of a dimer of WNV C protein [64]. The two subunits of the dimer are colored in cyan and blue respectively.

When the genetic stability of nucleotide substitution A103G was analyzed *in vivo* (Table 4), the presence of the nucleotide substitution was observed in the virus recovered from the brain of 2 out of the 4 dead mice analyzed (mice N.4.2, N.4.3). One of these mice (N.4.3) also displayed the accumulation of an additional nucleotide substitution responsible for amino acid replacement N15S in the prM protein. Interestingly, amino acid replacement N15S was located on the surface of the pr peptide (Fig. 8A) and involved a residue that constitutes a conserved N-linked glycosylation site [49]. Since the introduced S residue can also constitute a potential O-linked glycosylation site, this possibility was explored using NetOGlyc predictor server (http://www.cbs.dtu.dk/services/NetOGlyc/). However no significant O-linked glycosylation probability was found at this position for mutant pr, suggesting that N15S abolishes glycosylation at this position instead of altering the glycosylation pattern. The analysis of nucleotide sequences also revealed the appearance of a reversion to WT position in mice N.2.1, although this revertant had also selected two additional nucleotide substitutions responsible for amino acid replacements Q256L and L355M in the E glycoprotein. Both amino acid substitutions were located at the interfaz between domain I (DI) and DIII of the protein, (Figure 8A and B). These mutations were close to the single polypeptide linker that connects DIII with DI and could alter the major positional rearrangements in which participates this region during the virus life cycle [23], [25]. In addition to these viruses carrying mutations in the glycoproteins that constitute the external shell of the virion, the virus recovered from mice N.4.1 also displayed a reversion to WT position and selection of an additional amino acid replacement (I38L) in the C protein (Table 4) that was located at the end of α1 helix of C protein (Fig. 8C). Taken together, these results evidenced that reversion to WT phenotype or selection of additional nucleotide substitution were common among dead mice infected with Res virus, pointing to the fact that deleterious effect of nucleotide substitution, such as the A103G noticed *in vivo* (Fig. 7), can be compensated by the selection of viruses carrying amino acid substitutions in the structural proteins.

**Table 4:** Nucleotide sequence of virus isolated from the brain of dead mice infected with Res virus.

Mouse	Inoculum (PFU/mouse)	Death time (days p.i.)	Nucleotide substitutions found in the consensus sequence *^b^*			
			C	prM	M	E
N.2.1	10^2^	11				A1853T (**Q296L**) T2029A (**L355M**)
N.4.1	10^4^	7	A208C (**I38L**)			C1302T
N.4.2	10^4^	9	A103G (**K3E**)			
N.4.3	10^4^	10	A103G (**K3E**)	A509G (**N15S**)		

Brain from dead mice was homogenated and used to infect Vero cells. Viral RNA from infected cultures was extracted and amplified by RT-PCR and the nucleotide sequence of the genomic region encoding the structural proteins was determined. Nucleotide replacements found on the genomic sequence encoding WNV structural proteins were compared to WNV strain NY99-flamingo 382–99 [10]. Amino acid replacements are shown in bold within parentheses.

### A Mutation Located on the prM Protein Abolishes the Advantage Conferred by K3E Amino Acid Replacement of Res Virus

**Figure 9:**
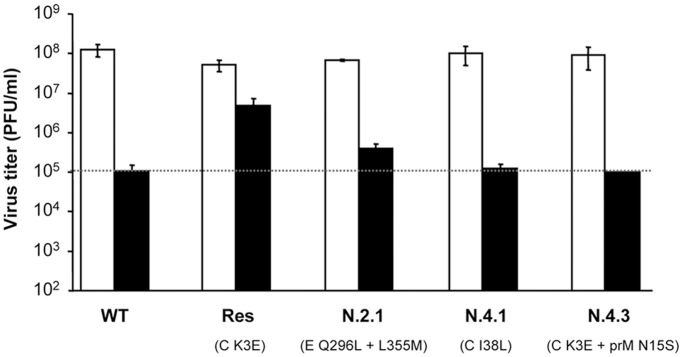
Analysis of the resistance to NH_4_Cl of viruses recovered from dead mice infected with Res virus. Vero cells were infected (MOI of 0.5 PFU/cell) in the absence (control) or in the presence of 25 mM NH_4_Cl with WT, Res, N.2.1, N.4.1 or N.4.3 virus. Amino acid replacements compared to WT are indicated into brackets for each virus (see also Table 4). Virus titers were determined 24 h.p.i. Dashed line indicates the virus titer shown by WT in the presence of NH_4_Cl. Error bars represent SD. The data presented are product of three independent experiments.

The resistance to NH_4_Cl of the viruses recovered from dead mice was tested for those viruses bearing additional amino acid substitutions (N.2.1, N.4.1 and N.4.3). To this end, cells were infected in the absence (control) or in the presence of NH_4_Cl and virus yield was determined by plaque assay (Fig. 9). All viruses analyzed reached similar virus titers in the absence of NH_4_Cl, indicating that these mutations did not confer major disadvantages under standard infections in tissue cultured cells. On the other hand, when NH_4_Cl was added to the infection medium, Res virus produced the higher virus yield, about one order of magnitude higher than that of WT, N.4.1 or N.4.3. In the case of N.2.1 virus, the inhibition exerted by NH_4_Cl was higher than for Res virus but not as marked as for WT, N.4.1 and N.4.3 viruses, thus displaying an intermediate phenotype. These results evidenced that revertant viruses N.2.1 and N.4.1 that had lost the amino acid replacement K3E at C protein found in Res virus, suffered from a reduction in their degree of resistance to NH_4_Cl. Interestingly, the virus N.4.3, which carried the additional substitution N15S on prM protein and that retained K3E amino acid replacement on C protein, grew at similar levels than WT in the presence of NH_4_Cl. This strongly suggests that mutation N15S found in the prM protein of this virus exhibited a compensatory activity that abolished the advantage conferred by K3E amino acid replacement on the C protein of Res virus under impaired organelle acidification.

## Discussion

In this report, the isolation and characterization of a WNV mutant with increased resistance to the acidotropic compound NH_4_Cl has been described. NH_4_Cl acts through ammonia diffusing into the cell [50]. At neutral pH, weak-base amines are uncharged and can diffuse across cellular membranes if they are sufficiently lipophylic. When diffused into acidic membrane compartments within cells, they become protonated. In their protonated form, the amines are less lipophylic and consequently they accumulate within acidified compartments and, thereby, raise the internal pH [50]. On the contrary, other inhibitors of acidification, as concanamycin A, block the function of the vacuolar ATPases, the enzymes that pump protons inside acidic organelles [43]. The WNV variant here selected displayed increased resistance to both inhibitors of acidification, showing that the viral mechanism to escape from inhibition of acidification exerted by NH_4_Cl was also valid to escape from concanamycin A. This indicates that the mutant was resistant to inhibition of acidification regardless of the method used to achieve it.

When the molecular basis of the increase in resistance to acidotropic compounds was addressed, it was shown that the resistant virus carried a single amino acid substitution (K3E) on the C protein. The nucleotide substitution (A103G) responsible for this amino acid replacement was introduced into an infectious clone and the phenotype was reproduced, thus confirming that this single amino acid substitution was sufficient to increase resistance to acidotropic compounds. In contrast to other flavivirus mutants with altered acidic pH requirements, which displayed amino acid substitutions on the external glycoproteins of the virion, E or M (see Introduction), the amino acid replacement here reported was located in the core of the virion, which is enveloped by the lipid bilayer. Considering that the WNV C protein has been proposed to be a determinant of virulence [51], and that single point mutations can either enhance [52] or decrease WNV virulence [53], it is not surprising that when the effect of K3E amino acid replacement was analyzed *in vivo* using the murine model, a major reduction of the infectivity *in vivo* was noticed. This can be derived from alterations of functions of basic amino acids inside this region during the multiple steps of the WNV infectious cycle (see below).

The WNV C protein is a multifunctional protein that can be found inside infected cells in both the cytoplasm and the nucleus [54], and has been implicated in multiple aspects of WNV infectious cycle including viral replication [55], induction of apoptosis [56], or disruption of epithelial barrier helping to virus dissemination [57]. Indeed, the interaction of the C protein of WNV with a growing variety of distinct cellular partners has been reported. These include Hsp70 [58], Jab1 [54], I_2_^PP2A^ [59], HDM2 [56], importin α/β [60], Sec3 [61], MKRN1 [62], or DDX56 [63]. The mature WNV C protein (105 amino acids) is produced by the cleavage of the hydrophobic C-terminus of the protein [1], [64]. The protein forms dimers that associate to form tetramers, which exhibit highly positively charged surfaces that are presumed to interact with the negatively charged RNA [64]. In fact, the presence of positively charged residues within the disordered regions of both the N and the C-terminus of the mature C protein has been associated to their ability to interact with nucleic acids [45], [65]. Nucleic acid binding properties of the C protein are important for RNA packaging in the nucleocapsid, and the lack of a well-formed protein shell in the nucleocapsid suggests that the basic C protein functions like a histone [64], [66]. In addition to this, and due to its RNA binding ability, the C protein promotes viral replication by helping RNA rearrangements and by contributing to RNA cyclization [45], [55]. The amino acid replacement found on Res virus (K3E) was located at the N-terminus of the protein, being part of a conserved epitope among the Japanese encephalitis serocomplex [67], and involved the change of one basic residue for an acidic one. This change of electric charge could carry alterations on the C functions involving RNA binding and/or interactions with cellular partners. According with this hypothesis, comparative genomic analysis of the C sequences from 486 strains revealed that mutations changing basic to acidic residues are very infrequent in WNV, and showed that this position in the WNV C protein was always occupied by a basic residue. In the case of Dengue virus, the mature C protein has been involved in virion morphogenesis due to its ability to interact with lipid droplets through its central hydrophobic residues [68], and the basic residues of the N-terminus of the C protein have been proved to be essential for viral particle formation [69]. Agreeing with this data, a role of the WNV C protein during the assembly of infectious particles has also been recently assessed [63]. The mutant here described showed an increase on the release of infectious particles, upon addition of NH_4_Cl or concanamycin A, compared to the WT. Interestingly, this phenomenon was not observed under standard culture conditions, or upon the addition of an inhibitor of furin-mediated maturation, suggesting that it takes place only when acidification is inhibited. Supporting this hypothesis, a similar inhibition of prM cleavage derived from NH_4_Cl treatment between WT and mutant viruses was observed. Results from in vitro furin cleavage of virions produced in the presence of NH_4_Cl pointed that acidic pH was also required for furin-mediated maturation of Res virus as well as for WT virus, thus, suggesting that, at neutral pH, Res virus does not undergo structural changes associated to virus maturation that expose the furin cleavage site.

Exposure of WNV particles to acidic pH in the absence of target membranes results in a loss of fusion capacity and viral inactivation [32], [44], [70], [71]. Conversely, flavivirus immature particles are deficient on membrane fusion and hence more resistant to acid-inactivation [39], [72]. As conformational rearrangements following acid exposure in solution resemble those induced inside endosomes that provide the driving force for membrane fusion [73], acid-inactivation analyses have been proven useful to analyze conformational rearrangements induced by acidic pH [32], [35], [44]. In the present report, no differences were observed between WT and Res WNV produced under normal conditions, suggesting that fusion pH of mature particles was similar for both viruses. However when a parallel analysis was carried with the viruses grown in the presence of NH_4_Cl (which are enriched in immature particles), Res WNV was more sensitive to acid-induced inactivation than WT, pointing to a better fusion competence of these particles. This phenomenon would constitute an advantage under conditions of impaired endosome acidification induced by NH_4_Cl or concanamycin A, explaining the molecular basis of resistance to inhibitors of organelle acidification. Indeed, increase in the sensitivity to acid-induced inactivation or displacement of fusion pH threshold to less acidic values is a well documented mechanism that reduces the sensitivity to drugs that impair endosome acidification [31], [74]. These findings are compatible with the observation that Res virus was able to produce more infectious (hence fusion-competent) particles/genomic RNA under conditions of impaired organelle acidification.

Several hypotheses can explain the molecular basis of this behaviour. For instance, alterations on the nucleocapsid could induce structural perturbations to the immature particles that facilitate conformational rearrangements triggered by acidic-pH, hence increasing their fusion competence and infectivity. However, this seems unlikely, since the furin cleavage site, whose exposure is associated to acid-induced conformational rearrangements associated to viral maturation [14], [39], was not exposed in Res virus grown in the presence of NH_4_Cl. An alternative non-excluding hypothesis is that the mutation in the capsid, located within a region putatively involved in RNA-binding, could lead to a more unstable virus, therefore, viral particles maybe able to release the genome more efficiently in the next cycle of infection even though the virus population consists mainly of immature virus. In any case, these rearrangements or alterations of the mechanical properties of the virion should take place without an increase in prM cleavage, since, at the level of resolution of our observations, the amount of M in Res particles grown in the presence of NH_4_Cl did not appear to be different from that of WT particles produced under similar conditions. This might imply that the increase in infectivity was not related to an increase in the degree of maturation of viral particles. In fact, several studies have shown that prM is present on infectious virions [17], [72], [75]. Thus, as recently reviewed [25], the infectivity of flaviviruses and its mechanistic is more probably a multifactorial process and, whereas the proteolytic processing of prM is a necessary step in the virus life cycle, complete maturation is not required for infectivity or fusion activity, so partially matured particles, heterogeneous in residual prM content and in arrangement of E proteins on the virion, can be infectious under certain conditions. Consisting with this view, the mutant WNV produced in the presence of NH_4_Cl exhibited increased infectivity than WT virus without showing any noticeable effect on the degree of maturation of viral particles, specifically in their M content or the need for acidic pH during in vitro treatment with furin.

Although it is not obvious to explain how a mutation located on the internal capsid protein can lead to alterations on the fusion-competence of virions, the interaction between capsid protein and surface glycoproteins is well documented for the alphaviruses [76], [77], [78], which share multiple features with flaviviruses related to its dependence on pH for membrane fusion [23]. Furthermore, it has been proposed that acid-induced rearrangements of alphavirus fusion glycoproteins might also cause a reorganization of the capsid-binding internal glycoprotein tails altering the nucleocapsid structure [79], a finding that evidence the crosstalk between components located in both sides of the viral envelope. Even more, a detailed analysis of the structure of immature flavivirus particles reveals that contacts between the nucleocapsid and the lipid bilayer can occur [80], supporting the notion of a crosstalk between the nucleocapsid and the external protein shell of the virion that directly interact with the acidic pH of the environment.

The selection of additional mutations on the structural proteins of virus isolated from dead mice infected with Res virus also points to the existence of a genetic connection between the internal C protein and the external virion proteins prM and E, supporting the notion of a crosstalk between the capsid protein and the surface glycoproteins. Indeed, the analysis of the genetic relationships between WNV proteins has revealed unexpected interactions [81]. When the resistance to NH_4_Cl of viruses recovered from dead mice was tested, it was observed that loss of K3E amino acid replacement was associated to a reduction on the degree of resistance to NH_4_Cl. Interestingly, the virus N.4.3 that carried the amino acid replacement N15S in the prM and conserved the K3E at the C protein, did not show any noticeable degree of resistance to NH_4_Cl. In this way, this replacement counteracted the advantage conferred by the K3E amino acid replacement on the C protein of the Res virus. The molecular basis of this compensatory effect are difficult to explain at this moment, however it is well known that the prM facilitates the folding and trafficking of the E protein during virus biogenesis and that removal of pr peptides from the virion primes the viral particle in their mature, metastable structural state, ready for the low-pH triggered fusion [25]. Therefore, changes in this region could have multiple effects during virus maturation and/or entry. The N15S replacement is located at the conserved N-linked glycosylation site of the prM [49], thus probably altering its glycosylation. The impairment of the glycosylation of this protein by introduction of the N15Q amino acid replacement has been shown to reduce secretion of WNV particles, although with modest effect on their infectivity, and thus, it has been hypothesized that the glycosylation of the prM assists protein folding at the endoplasmic reticulum rather than virus infection [49].

Assessment of whether an amino acid replacement on the virion core, surrounded by a lipid envelope, can contribute to evasion from acidotropic drugs, whose effect is supposed to target the external virion proteins, which are accessible to pH variations, is not an easy task. In this way, the genetic connection between the mechanisms of escape reported for this mutant and its relationship with acidic pH organelles invites to revisit the role of the C protein during entry and assembly of WNV and other related flaviviruses.

## Supporting Information

Figure S1 **Prediction of disordered and hydrophobic regions of the WNV C protein.** (**A**) Disorder disposition of the amino acid residues of the WNV C protein strain NY99-flamingo 382–99 was calculated with the meta-predictor PONDR-FIT™ [83]. (**B**) Prediction of hydrophobic regions of the WNV C protein performed with TMHMM [84]. White arrow points to the position mutated that increased resistance to NH_4_Cl. Black arrowhead indicates the site of maturation cleavage [64].
(TIF)

Figure S2 **Multiple sequence alignment of the flavivirus C proteins.** Multiple alignment was performed with T-COFFEE [85]. GenBank accessions: WNV (AF196835.2), JEV, Japanese encephalitis virus (NC_001437.1); MVEV, Murray Valley encephalitis virus (NC_000943.1); USUV, Usutu virus (NC_006551.1); SLEV, St. Louis encephalitis virus (AEN02430.1); DENV, Dengue virus (ACF49259.1, ACW82869.1); YFV, Yellow Fever virus (ACN41908.1). Basic and acid residues are highlighted. White arrow points to the position mutated that increased resistance to NH_4_Cl. Black arrowhead indicates the site of maturation cleavage [64].
(TIF)
