# Supplementary figures and images for: A Single Amino Acid Substitution in the Core Protein of West Nile Virus Increases Resistance to Acidotropic Compounds
Source: PLoS One. 2013 Jul 18;8(7):e69479. doi: 10.1371/journal.pone.0069479 (PMC3715472; doi:10.1371/journal.pone.0069479)

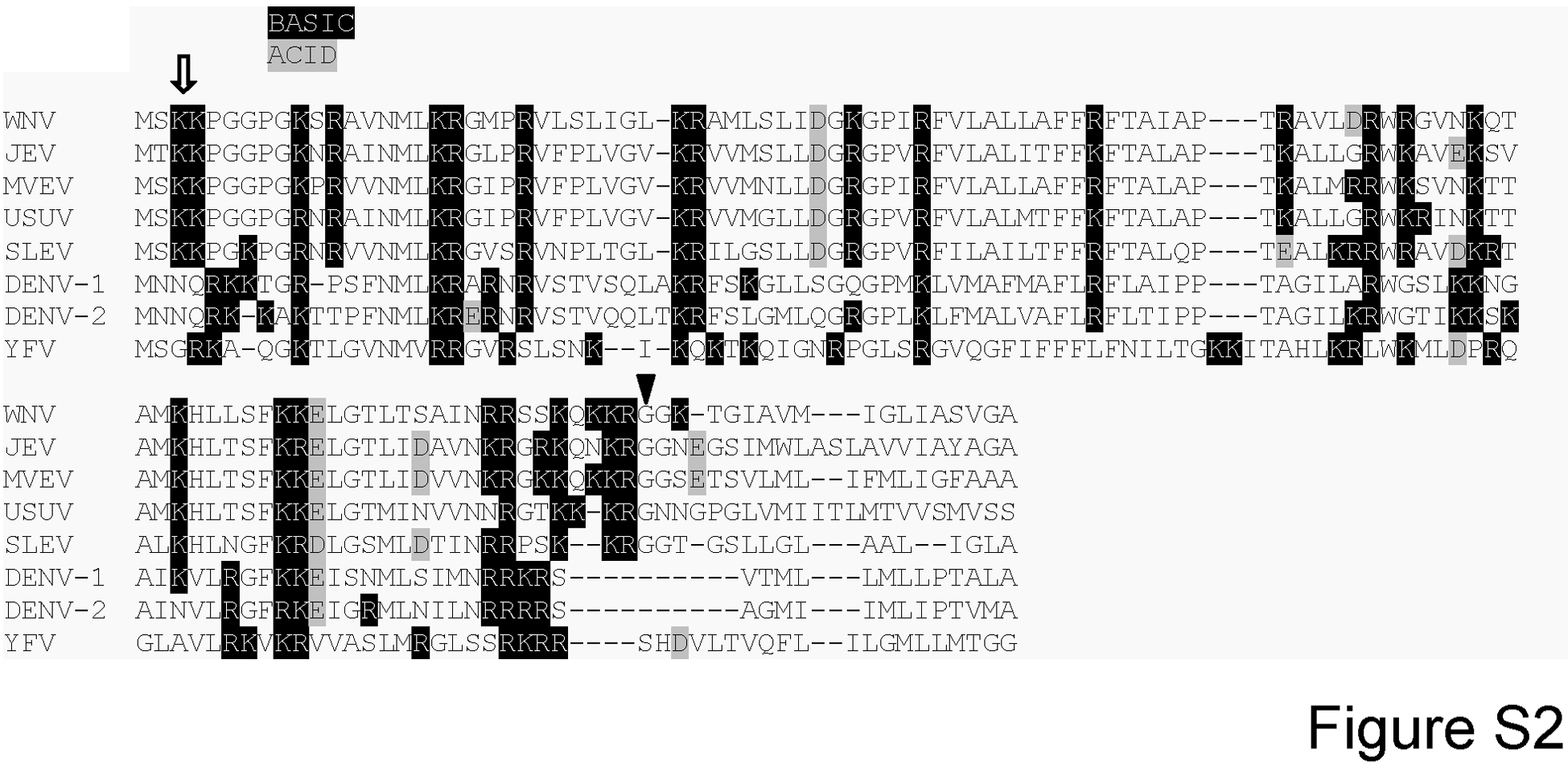

Supplement: Figure S2 — Multiple sequence alignment of the flavivirus C proteins. Multiple alignment was performed with T-COFFEE [85]. GenBank accessions: WNV (AF196835.2), JEV, Japanese encephalitis virus (NC_001437.1); MVEV, Murray Valley encephalitis virus (NC_000943.1); USUV, Usutu virus (NC_006551.1); SLEV, St. Louis encephalitis virus (AEN02430.1); DENV, Dengue virus (ACF49259.1, ACW82869.1); YFV, Yellow Fever virus (ACN41908.1). Basic and acid residues are highlighted. White arrow points to the position mutated that increased resistance to NH4Cl. Black arrowhead indicates the site of maturation cleavage [64]. (TIF) [file pone.0069479.s002.tif]
